# Inactivation of African swine fever virus inoculated in liquid plasma by spray drying and storage for 14 days at 4°C or 20°C

**DOI:** 10.1371/journal.pone.0290395

**Published:** 2023-08-22

**Authors:** Elena Blázquez, Joan Pujols, Joaquim Segalés, Núria Navarro, Carmen Rodríguez, Jesús Ródenas, Javier Polo

**Affiliations:** 1 IRTA, Centre de Recerca en Sanitat Animal (CReSA), Barcelona, Spain; 2 APC Europe, S.L.U. Granollers, Granollers, Spain; 3 OIE Collaborating Centre for Emerging and Re-Emerging Pig Diseases in Europe, IRTA-CReSA, Barcelona, Spain; 4 Departament de Sanitat i Anatomia Animals, Universitat Autónoma de Barcelona (UAB), Barcelona, Spain; 5 APC LLC, Ankeny, IA, United States of America; University of South Florida, UNITED STATES

## Abstract

African swine fever virus (ASFV) is a dsDNA virus that can cause high mortality in pigs of all ages. Spray-dried porcine plasma (SDPP) is a highly digestible ingredient used in feed because it benefits performance, gut function and immunity. The objectives were to test if the spray-drying (SD) conditions along with post-drying storage of product for 14 days can inactivate ASFV inoculated in liquid plasma. Fresh liquid porcine plasma was inoculated with ASFV (BA71V) to a final concentration of 10^5.18 ±0.08^ TCID_50_/mL of liquid plasma. Triplicate 2-L samples of spiked plasma were SD in a lab drier set at an outlet temperature of 80°C or 71°C. The final dried samples were stored at 4°C or 20°C for 14 d. Liquid and SD samples were analyzed for ASFV infectivity in two mirror 24-well plaques containing VERO cells monolayers. Wells were inoculated with different dilutions of SDPP dissolved 1:9 in PBS. One plaque was immediately frozen at -80°C and the other was incubated at 37°C for 3 d. Each dilution was replicated 9 times. After incubation both plaques were analyzed for ASFV by qRT-PCR. Results indicated that the SD process inactivated between 3.2 to 4.2 Logs ASFV TCID_50_/mL and 2.53 to 2.75 Logs TCID_50_/mL when the outlet temperature were 80°C and 71°C respectively. All SD samples stored at 4°C or 20°C for 14 d were absent of infectious ASFV. The combination of SD and post drying storage at both temperatures for 14 d was able to inactive >5.18 ±0.08 Log_10_ of ASFV inoculated in liquid porcine plasma, demonstrating that the manufacturing process for SDPP can be considered safe regarding ASFV.

## Introduction

Spray-dried porcine plasma (SDPP) is a highly digestible, high-protein ingredient that is widely used in feed because it benefits growth performance, feed efficiency, intestinal integrity, and immune parameters [1–3].

The manufacturing process of SDPP begins with the collection of blood from healthy animals declared fit for slaughter for human consumption by the veterinary authorities. Blood is collected into containers with anticoagulant, chilled, and centrifuged to separate plasma. Liquid plasma is then concentrated and spray-dried at 80°C throughout its substance to produce SDPP [3, 4]. Spray-drying involves very rapid desiccation of the liquid [5], results in a dried powder with low moisture (< 9%) and very low water activity (aw < 0.6), which has a detrimental effect on bacteria and virus survivability, as has been shown for multiple bacteria and viruses of interest in swine industry [6–12]. Furthermore, some microorganisms, especially bacteria and enveloped viruses, are not able to survive for a prolonged time in dried materials like SDPP with very low water activity [3, 5, 8, 12, 13]. Therefore, a minimum post-drying period of 14 days at 20°C has been established as processing standards of the European Animal Protein Association (EAPA) (EAPA Code of Practice, https://www.eapa.biz/quality-safety) and been adopted by most SDPP manufacturers.

*African swine fever virus* (ASFV) is an enveloped dsDNA virus belonging to the *Asfarviridae* family [14] that can cause high mortality in pigs of all ages. In 2007, a virulent ASFV strain (genotype II) appeared in Georgia causing mortality rates up to 100%, and from there spread across the Caucasus region into the Russian Federation, Easter Europe, and other West European countries (Belgium, Germany, Italy, Greece and Czech Republic). Subsequently, from 2018 onwards, ASFV further spread to China, South-East Asia, and more recently, in the Dominican Republic and Haiti, where millions of animals have succumbed to the disease [15, 16]. At global level there is no commercial vaccine available to fight against the ASF pandemic; however, recent results with recombinant live attenuated viruses provided hope for a safe and effective vaccine against ASFV as demonstrated by the launching of the first commercial vaccine against ASFV in Vietnam. This vaccine product is based on a recombinant deletion mutant lacking the I177L gene from the Georgia 2007 ASFV isolate [15–17].

The prevalence of African Swine Fever (ASF) has increased concerns that feed ingredients could represent a risk factor for spreading ASF. Spray-drying has shown an ASFV inactivation capacity of 4.11 ± 0.20 Log_10_ TCID_50_/mL [18] resulting in the recognition of SDPP as a negligible risk for transmitting the disease [19]. However, other studies are necessary to characterize the inactivation capacity of other processing steps included in the manufacturing process of SDPP, such as the post-processing storage period at 20°C.

The objective of the present study was to determine the level of ASFV inactivation in liquid plasma artificially contaminated with ASFV by the safety steps involved in commercial SDPP production including spray-drying in combination with storage for 14 days at different temperatures.

## Material and methods

### African swine fever virus

ASFV strain Badajoz-71 adapted to Vero cells (ASFV-BA71-V) [20] was provided by Dr. María Luisa Salas from Centro de Biología Molecular Severo Ochoa (CBMSO), Madrid, Spain. The virus was propagated in Vero cells (ATCC CCL-81) grown in DMEM (ThermoFisher, Waltham, MA, USA) supplemented with 10% FBS (BioWest, Florida, USA), 200 mM glutamine (ThermoFisher), penicillin 100 UI/mL (ThermoFisher), streptomycin 100 μg/mL (ThermoFisher), and nystatin 40 UI/mL (Sigma-Aldrich, Missouri, USA). Viral stock solution was produced in this way in successive passages obtaining a final viral titer of 10^6.9^ TCID_50_/mL measured by immunoperoxidase monolayer assay (IPMA).

### Plasma

The plasma used for this experiment was obtained from pigs slaughtered in European porcine abattoir facilities from animals inspected and approved as fit for slaughter for human consumption by official veterinary authorities. Blood was collected in stainless steel containers, with an anticoagulant (sodium tripolyphosphate), refrigerated and transported to the APC Europe facilities (APC-Europe S.L.U., Granollers, Spain). Plasma was separated in less than 24 hours from collection by commercial centrifugation. Eight liters of fresh plasma were UV-C irradiated at 10000 J/L for this study using the UV-C SurePure SP-1 device as previously described [21, 22] to inactivate any microorganisms before the ASFV inoculation. A 10 mL sample of the 8-L batch was stored at –80°C and later used as a control to determine ASFV antibodies (INgezim PPA COMPAC, INGENASA; Madrid, Spain) or genome by real time PCR (qRT-PCR) analysis [23].

### ASFV spray-drying inactivation

Three 2-L batches of UV-C irradiated liquid porcine plasma were used for the study. From each batch, 200 mL of the 2 L were collected and stored at –80°C for later analysis. The remaining 1800 mL of liquid plasma per batch were inoculated with 200 mL of ASFV-BA71-V stock solution (10^6.9^ TCID_50_/mL) achieving a viral theoretical titer of 10^5.9^ TCID_50_/mL.

The spray-drying process was conducted using a laboratory spray-drier (Büchi Mini Spray Dryer B-290, Büchi Labortechnik, Switzerland) simulating industrial process conditions. The laboratory spray-drier was set to an inlet air temperature of 200 ± 5°C and outlet air temperature set at 80 ± 1°C or at 71 ± 1°C as previously described [10]. Airflow through the column and the suspension flow to the nozzle was set at 45 m^3^ h^-1^ (at 20°C) and 0.2 lh^-1^, respectively. The airflow through the feed nozzle adjusted to 0.7 m^3^h^-1^ (at 20°C). Residence time was estimated to be 0.41 s.

Before processing and collecting dried samples for analysis, the lab spray-dryer processing parameters were stabilized by running it with non-inoculated UV-C irradiated liquid porcine plasma at the target temperature for 10 min. After this time, the sample collector for the spray-dryer was replaced with a clean one and the previously collected powder was discarded. This preliminary drying time assures that the system was stabilized at the desired processing parameters before the ASFV inoculated plasma was dried and samples for analysis were collected.

**Fig 1:**
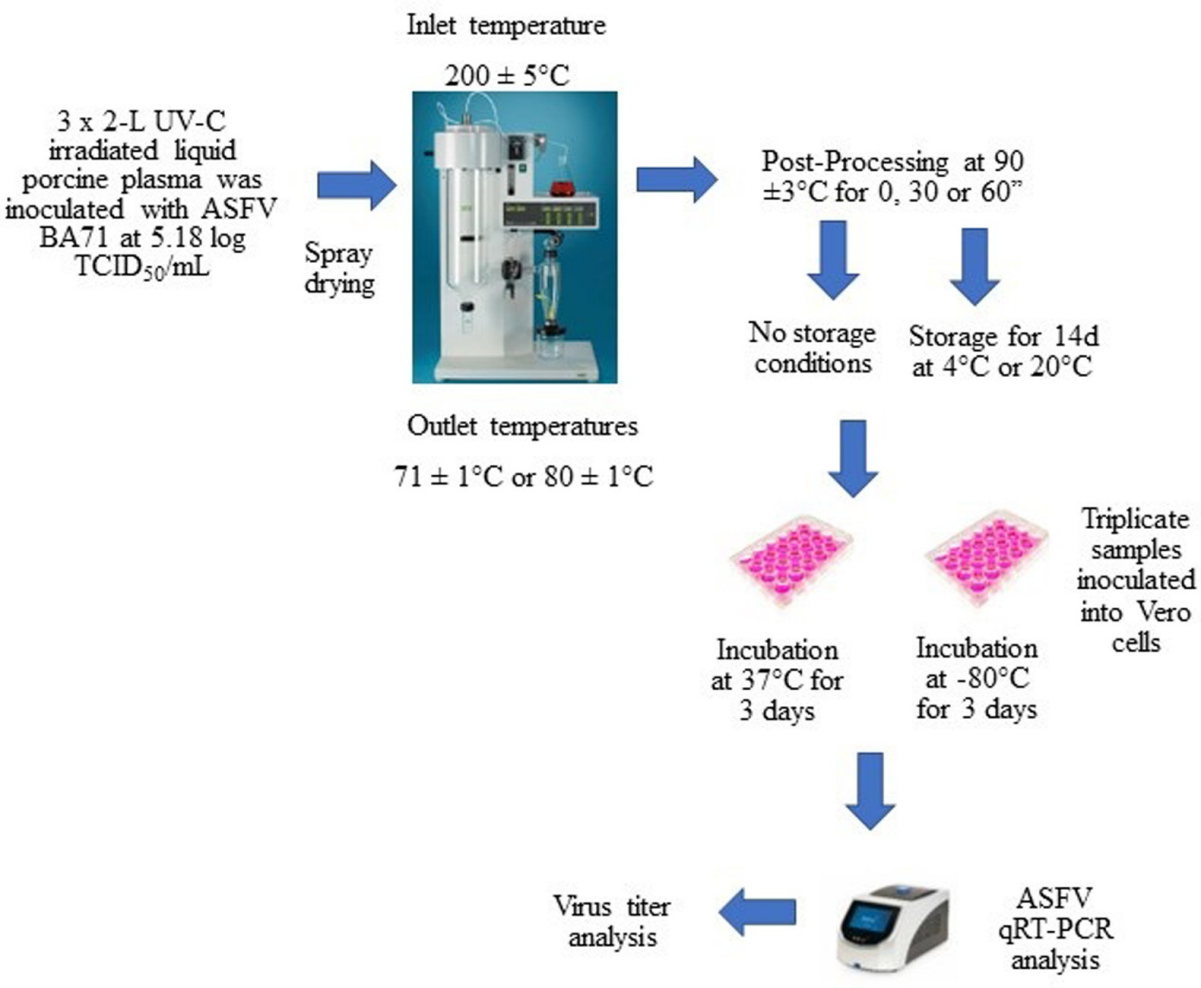
Scheme of the experiment.

Each dried inoculated sample was distributed in twenty-seven samples of 0.5 g in 0.5 cm glass tubes (inner diameter). Nine samples were obtained directly without receiving additional heat post drying. To simulate the longer retention time in commercial spray driers, an additional 9 samples were placed in a water bath at 90±3°C for 30 seconds (real temperature in the powder sample was around 70.4°C) and another nine samples for 60 seconds (real temperature in the powder sample was around 80.7°C). The temperature of the spray dried samples during the 30 s and 60 s post-drying heat treatment was monitored with temperature probes. In each simulation, three of these samples were analyzed directly, three samples were stored at 4°C for 14 days and another 3 samples at 20-22°C (room temperature) for 14 days (Fig 1).

### Sample analysis

Spray-dried samples were reconstituted by adding 4.5 mL of PBS to 0.5 g of plasma sample (ratio SDPP 1:9 PBS). A ten-fold dilution series was done from native samples to -3 dilution. Each dilution was inoculated in 9 wells, obtaining 9 replicates of the same sample. This procedure was done by duplicate to obtain two identical mirror 24 wells plates. Of these two identical plates, one of them was immediately frozen at -80°C and the other one was incubated at 37°C for 3 days. After the 3-day incubation period, the plates were frozen and thawed and the frozen plates were thawed also.

**Fig 2:**
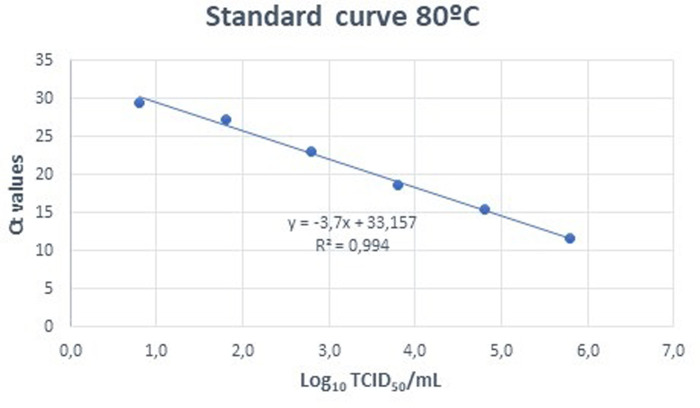
Standard curve of ASFV Log TCID_50_/mL vs Ct for samples spray-dried at 80°C.

**Fig 3:**
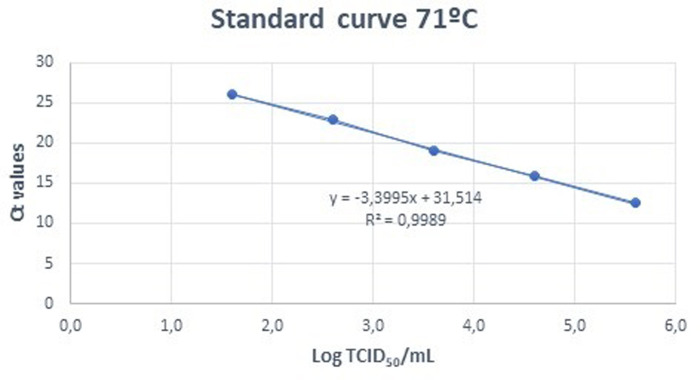
Standard curve of ASFV Log TCID_50_/mL vs Ct for samples spray-dried at 71°C.

Both mirror plates were analyzed for presence of ASFV by qRT-PCR [23]. From the 9 inoculated wells for each dilution, 100 µL were taken from three wells and mixed to perform a single extraction from those 3 wells. In this way, from the 9 initial wells, 3 DNA extractions (INDICAL, INDICAL BIOSCIENCE, Leipzig, Germany) were performed for each dilution. Subsequently, these three extractions were analyzed by qRT-PCR to obtain three replicates of each dilution. We compared the difference between the Ct obtained in the frozen plate and the plate incubated at 37°C, assuming that a difference of Ct in the incubated plate compared to the frozen plate implied viral replication in the Vero cell culture [24]. The reduction factor was calculated as the difference between the virus titer detected in the inoculated material at start of the experiment and the titer or absence of replication detected in the final sample after processing. Values were calculated as the reduction in Logs TCID_50_/mL including standard error. Standard curves were established at each outlet temperature by regressing Log_10_ TCID_50_/mL SDPP on CT results (Figs 2 and 3).

## Results

The spray-drying process at an outlet temperature of 80°C throughout its substance inactivated ASFV between 3.2±0.07 to 4.2±0.07 Logs TCID_50_/mL. When the outlet temperature was reduced to 71°C, ASFV inactivation was reduced to 2.53±0.12 to 2.75±0.04 Logs TCID_50_/mL (Table 1).

**Table 1:** African swine fever virus (ASFV) inactivation during spray-drying at different outlet temperatures and residence times and further storage at either 4°C or 20°C for 14 d. Initial ASFV inoculation in liquid plasma was 10^5.18^ ±0.08 TCID_50_/mL.

Conditions	SDPP (TCID_50_/mL)	LRVs during SD	Storage of SDPP (4°C or 20°C) for 14days (TCID_50_/mL)
		(TCID_50_/mL)	
SD at 80°C; No post treatment	1.94±0.07	3.23±0.07	Negative
SD at 80°C; RT 30 sec at 80°C	1.35±0.06	3.82±0.07	Negative
SD at 80°C; RT 60 sec at 80°C	0.97±0.07	4.20±0.07	Negative
SD at 71°C; No post treatment	2.59±0.14	2.58±0.14	Negative
SD at 71°C; RT 30 sec at 80°C	2.64±0.12	2.53±0.12	Negative
SD at 71°C; RT 60 sec at 80°C	2.42±0.04	2.75±0.04	Negative

SD = spray-drying; RT = residence time; SDPP = spray-dried porcine plasma; LRVs = Log_10_ reduction values

The ASFV inoculated spray-dried samples obtained from both outlet temperatures with different residence times (0 to 60 seconds) when stored either at 4°C or 20°C for 14 days were completely inactivated (Table 1). Our data suggested that both storage temperatures after the spray-drying process inactivated at least an additional 2.65±0.08 Log of ASFV.

## Discussion

Spray-dried plasma (SDP) is a functional protein source that significantly improves daily gain, feed intake, production efficiency and reduces post weaning lag [2, 25–29]. The manufacturing process of SDP involves several safety features as veterinary inspection at abattoir, pooling of plasma, spray-drying process and storage [3, 4]. Veterinary inspection is crucial to ensure that blood from only healthy animals slaughtered for human consumption is the exclusive source of raw material to be used for the manufacturing of blood products. However, veterinarian inspections might not find subclinical illnesses or early viremia/bacteremia, therefore, implementing, understanding, and validating the biosafety measures are therefore crucial for the manufacturing process of SDP.

Several viruses of concern to the swine industry, including the *Porcine respiratory and reproductive syndrome virus* (PRRSV), *Pseudorabies virus* (PRV), and *Porcine epidemic diarrhea virus* (PEDV), have been shown to be effectively inactivated by spray-drying [9–12], as well as bacteria such as *Escherichia coli* or *Salmonella enterica* [7, 8].

The phenomena involved in spray-drying inactivation of these pathogens rely on the rapid desiccation [5], cytoplasmic membrane damage [30, 31], genetic material destruction, and inactivation of other proteins, including enzymes [31]. Under commercial conditions, during the spray-drying process, SDP is exposed to a minimum of 80°C throughout substance [3], a temperature that is recognized as effective to inactivate pathogens such as ASFV, CSFV, *Swine vesicular disease virus* and *Foot and mouth disease virus* (FMDV) in cooked meat products (“Council Directive 2002/99/EC of 16 December 2002). In the present study we tested the commercial outlet air temperature conditions of 80°C and 71°C. Under these conditions we observed higher ASFV inactivation at 80°C (range between 3.23–4.20 Log reduction Value (LRV)) than 71°C (range 2.53±0.12–2.75±0.04 LRV), results that agrees with publications indicating that pathogen inactivation during spray-drying is related to the inlet and outlet air temperatures, especially the outlet temperature [3, 5, 32]. These results also agree with the code of practice that established 80°C outlet temperature as the recommended drying temperature for commercial blood products intended for use in swine feedstuff (EAPA, 2022). In addition, the results confirm that the higher residence time of commercial driers (30 to 60 seconds) increases the inactivation of ASFV inoculated in liquid plasma compared to the residence time of laboratory driers (<1 sec), as demonstrated in previous publications that show higher residence time inside spray driers may increase the inactivation of microorganisms [5, 32].

In previous studies spray-drying at 80°C for 60 s, was able to inactivate 4.11 Log TCID50/mL of ASFV [15]. In the present study, we wanted to characterize the loss of viability that occurs naturally in the storage step of SDPP manufacturing process, comparing two different storage temperatures. Post-drying storage for 14 days at 4°C or 21±2°C resulted in complete inactivation of ASFV. The additional 1.95±0.08 Log_10_ TCID_50_/mL inactivation at 80°C or 2.65±0.08 Log_10_ TCID_50_/mL at 71°C partially agrees with results obtained by Fischer et al., 2021 [24], who achieved a complete inactivation of 5.7 HAD/ml after two weeks of storage at room temperature, but limited inactivation was achieved for SDPP spiked with ASFV and stored at 4°C. However, we observed a 2.65±0.08 Log_10_ TCID_50_/mL loss of viability when SDPP was stored at 4°C. The differing results between our study and theirs may be that in our study we inoculated ASFV into the liquid plasma before drying, whereas they spiked ASFV on previously dried porcine plasma. Therefore, the initial viral titer after spray drying in our study was lower than the initial viral titer of the spiked SDPP used in the study by Fischer et al. (2021). In addition, we started the storage period just after spray-drying of the liquid plasma without time for the virus to recover from the heat and desiccation effects associated with the spray-drying process. In contrast, the current results are consistent with the inactivation of 2 Log_10_ TCID_50_ observed by Dee et al., 2018 [33] over 30 days at room temperature.

Furthermore, in a previous study [18] it was demonstrated that the minimum infectious dose in feed mixed with ASFV contaminated liquid porcine plasma was higher than 5.0 Log_10_ TCID_50_/pig. Considering this minimum infective dose of ASFV in the feed, the proven inactivation capacity of the spray-drying process (> 4 log TCID_50_/mL) and the subsequent inactivation during post-drying storage (inactivation between 1.95 to 5.7 Log_10_ TCID_50_/mL) within two weeks at room temperature, the manufacturing process for SDPP has a high inactivation capacity against ASFV.

The overall results demonstrated that spray-dry process at 80°C or 71°C and the subsequent storage at room temperature for 14 days inactivates >5.18 ±0.08 Log_10_ TCID_50_/mL of ASFV result in a safe feed ingredient for use in swine feed.
